# E‐HCAP, a validated version of the cognitive assessment tool adapted to the Egyptian cultural and educational settings

**DOI:** 10.1002/alz70856_097497

**Published:** 2025-12-24

**Authors:** Mohamed Salama

**Affiliations:** ^1^ The American University in Cairo, Cairo, Egypt

## Abstract

The global burden of dementia underscores the urgent need for accessible, culturally relevant, and scientifically robust tools to assess cognitive health. Our study aimed to address this need by developing and validating innovative digital cognitive assessment tools within the Egyptian context. A cohort of 1,200 participants aged 55 years and older was recruited from eight diverse sites across Egypt, representing urban and rural communities with varying educational levels. This heterogeneity was integral to ensuring inclusivity and representativeness.

The Harmonized Cognitive Assessment Protocol (HCAP) served as the benchmark for cognitive evaluation. Alongside, we piloted two novel digital tools: one leveraging speech analysis to detect cognitive markers and another exploring olfactory recognition as an indicator of cognitive function. These tools were rigorously validated against the HCAP to assess their sensitivity and reliability. Detailed health profiles were collected to contextualize cognitive performance, focusing on the 12 modifiable risk factors for dementia, offering insights into the intersection of health determinants and cognitive aging.

To deepen our understanding of the biological underpinnings of cognitive decline, venous blood samples were collected from all participants for biomarker analysis. By integrating digital innovation with established protocols and biological measures, this study provides a comprehensive framework for cognitive assessment in resource‐limited settings and highlights the human dimensions of aging, vulnerability, and resilience. Our findings aim to inform public health strategies and contribute to the equitable advancement of dementia research globally.

